# Glutamatergic neurometabolite levels in major depressive disorder: a systematic review and meta-analysis of proton magnetic resonance spectroscopy studies

**DOI:** 10.1038/s41380-018-0252-9

**Published:** 2018-10-12

**Authors:** Sho Moriguchi, Akihiro Takamiya, Yoshihiro Noda, Nobuyuki Horita, Masataka Wada, Sakiko Tsugawa, Eric Plitman, Yasunori Sano, Ryosuke Tarumi, Muhammad ElSalhy, Nariko Katayama, Kamiyu Ogyu, Takahiro Miyazaki, Taishiro Kishimoto, Ariel Graff-Guerrero, Jeffrey H. Meyer, Daniel M. Blumberger, Zafiris J. Daskalakis, Masaru Mimura, Shinichiro Nakajima

**Affiliations:** 1 Department of Neuropsychiatry, Keio University School of Medicine, Tokyo, Japan; 2 Research Imaging Centre, Centre for Addiction and Mental Health, University of Toronto, Toronto, Canada; 3 Department of Pulmonology, Yokohama City University Graduate School of Medicine, Yokohama, Japan; 4 Temerty Centre for Therapeutic Brain Intervention, Centre for Addiction and Mental Health, Department of Psychiatry, University of Toronto, Toronto, Canada

**Keywords:** Depression, Neuroscience

## Abstract

Alterations in glutamatergic neurotransmission are implicated in the pathophysiology of depression, and the glutamatergic system represents a treatment target for depression. To summarize the nature of glutamatergic alterations in patients with depression, we conducted a meta-analysis of proton magnetic resonance (^1^H-MRS) spectroscopy studies examining levels of glutamate. We used the search terms: depress* AND (*MRS OR* “*magnetic resonance spectroscopy*”). The search was performed with MEDLINE, Embase, and PsycINFO. The inclusion criteria were ^1^H-MRS studies comparing levels of glutamate + glutamine (Glx), glutamate, or glutamine between patients with depression and healthy controls. Standardized mean differences (SMD) were calculated to assess group differences in the levels of glutamatergic neurometabolites. Forty-nine studies met the eligibility criteria, which included 1180 patients and 1066 healthy controls. There were significant decreases in Glx within the medial frontal cortex (SMD = −0.38; 95% CI, −0.69 to −0.07) in patients with depression compared with controls. Subanalyses revealed that there was a significant decrease in Glx in the medial frontal cortex in medicated patients with depression (SMD = −0.50; 95% CI, −0.80 to −0.20), but not in unmedicated patients (SMD = −0.27; 95% CI, −0.76 to 0.21) compared with controls. Overall, decreased levels of glutamatergic metabolites in the medial frontal cortex are linked with the pathophysiology of depression. These findings are in line with the hypothesis that depression may be associated with abnormal glutamatergic neurotransmission.

## Introduction

The heterogeneity of the illness features that characterize depression makes it difficult to elucidate the underlying pathology of the illness and its treatment. The glutamate hypothesis of depression was proposed in the 1990s, when antagonists of the *N*-methyl-d-aspartate (NMDA) receptor, an ionotropic glutamate receptor, were found to possess antidepressant-like mechanisms of action in mice [1]. Furthermore, infusion of low-dose ketamine, which is an NMDA receptor antagonist, is associated with robust decreases in depressive symptoms in depressed patients [2].

Recent data suggest that glutamatergic dysfunction is involved in the biological mechanisms underlying depression [3]. For example, positron emission tomography (PET) studies have reported reduced metabotropic glutamate receptor subtype 5 density in patients with depression, which was further substantiated by a post-mortem study [4]. In addition, animal model studies have also demonstrated that depressive-like behaviors are associated with alterations in cortical glutamate [5–7]. Furthermore, a recent meta-analysis showed that ketamine has rapid antidepressant effects in depressed patients compared with placebo [8]. As a result, glutamatergic neurometabolites have garnered increasing interest in terms of their role in the underlying pathophysiology of depression.

To date, several proton magnetic resonance spectroscopy (^1^H-MRS) studies have examined regional levels of glutamatergic metabolites in patients with depression compared with controls. However, findings are inconsistent across studies, which report increases [8], no differences [9, 10], or decreases [11–13] in glutamatergic neurometabolite levels in patients with depression across a variety of brain regions. These differences may be due to differences in regions of interest (ROIs), MRS methodologies, stages or severities of illness, or medications (e.g., antidepressant treatments). A recent meta-analysis noted that glutamate levels were lower within the anterior cingulate cortex (ACC) of patients with depression compared with controls [14]; of note, this meta-analysis included 16 studies published until 2010 and consisted of 281 patients and 301 controls. There were some limitations in this meta-analysis, as several of the included studies had all of the ROIs merged into one [14]. Furthermore, another recent meta-analysis reported that glutamine + glutamate (Glx) levels were decreased in the prefrontal cortex (PFC) in patients with depression compared with controls, whereas no significant difference was found in terms of glutamate levels between the two groups [15]. This meta-analysis also noted that reductions in Glx levels within the PFC were related to the number of failed antidepressant treatment trials. Specifically, this meta-analysis focused exclusively on glutamate and Glx in the PFC, and included 17 studies published until 2014, totaling 363 patients and 306 controls [15]. Notably, the inclusion of more recent reports would allow the meta-analysis of data from specific brain regions in patients with depression. Importantly, 22 papers, which is more than double the number of studies included in past meta-analyses, have been published since these meta-analyses.

Therefore, we conducted a systematic review and meta-analysis to compare the levels of specific regional glutamatergic neurometabolites; we aimed to do so in a comprehensive fashion, including the most recent studies on the topic. Based on previous meta-analyses, we hypothesized that Glx levels in the medial PFC (mPFC) would be decreased in patients with depression compared with controls [14, 15]. We also explored the influences of age, sex, symptom severity, and antidepressant treatment on group differences in the levels of regional glutamatergic neurometabolites.

## Methods

### Protocol registration

The full protocol was uploaded to the International Prospective Register of Systematic Reviews website (CRD42017079668). We have followed the Preferred Reporting Items for Systematic Reviews and Meta-Analyses (PRISMA) statement [16].

### Study search

We used the search terms: depress* AND (*MRS OR* “*magnetic resonance spectroscopy*”). The search was performed with MEDLINE (1946 to October 2017), Embase (1947 to October 2017), and PsycINFO (1806 to October 2017). The searches were rerun just before the final analyses and further studies were retrieved for inclusion in March 2018.

A hand search was conducted by SM, AT, and SN. Candidate articles were independently screened and scrutinized by these authors. Discrepancies in study selection were resolved by discussion among the three authors.

### Data extraction

Any data concerning a fundamental description of each study and data related to the outcomes described below were independently extracted by SM and AT. We have extracted any data regardless of the definitions of the primary/secondary endpoints of each original study. The extracted data were cross-checked and discrepancies were resolved by discussion between the two authors. If different publications reported data from the same population, we included data from the publication with the larger sample size. When studies did not report data, we e-mailed the authors to obtain the data.

### Inclusion criteria

#### Publication type

Any English, full-length, or short articles were included, whereas non-English articles and conference abstracts were excluded.

#### Study design

We included cross-sectional studies and randomized control studies with MRS data in both patients with depression and healthy controls.

#### Patients

Studies were included if: (1) patients met the Diagnostic and Statistical Manual of Mental Disorders (DSM), 3rd, 4th, or 5th edition criteria for major depressive disorder without bipolar disorder, the International Classification of Disease diagnostic (ICD) criteria for major depressive disorder without bipolar disorder, or consensus expert evaluation confirmed the diagnosis of depression without bipolar disorder; (2) we included all age range from pediatric to senior subjects; (3) authors compared glutamate, glutamine, or glutamine + glutamate (Glx) levels in the brains of patients with depression and subjects without depression using ^1^H-MRS; (4) authors included at least three subjects in each group; and (5) data were sufficient and appropriate to obtain mean differences between groups. In contrast, studies were excluded if they did not present data exclusively from patients with depression.

### Quality assessment

The quality of the original studies was assessed using the Newcastle-Ottawa Quality Assessment Scale after arranging it for a cross-sectional study design [17]. This scale assigns four and two points for patient selection and comparability, respectively. Six points indicated the highest quality, whereas zero points indicates the lowest quality.

### Primary outcomes

The primary outcome was Glx levels. The secondary outcomes were glutamate and glutamine levels. The ROIs were as follows: (1) the mPFC, including both the mPFC and ACC since their ROIs often overlap; (2) the dorsolateral PFC (DLPFC); (3) the thalamus; (4) the medial temporal lobe (mTemp), including the hippocampus and para hippocampus; and (5) the occipital cortex. When data from bilateral lobes were reported separately, the left lobe was used because the left lobe was examined in most studies.

### Statistical analyses

All continuous primary and secondary outcomes were compared between patients and controls using the standardized mean difference (SMD). SMD and two-sided 95% confidence intervals (CIs) were chosen as the summary statistic for the meta-analysis. Interpretation of the magnitude of the SMD was as follows: small, SMD = 0.2; medium, SMD = 0.5; and large, SMD = 0.8. The calculation of SMD was conducted using Review Manager ver. 5.3 (Cochrane Collaboration, Oxford, UK). We conducted a meta-analysis for each metabolite in each ROI that included four studies or more. The heterogeneity among original studies and subgroups was evaluated using the *I*^2^ statistic, whereby *I*^2^ = 0% indicated no heterogeneity, 0% < *I*^2^ < 30% indicated the least heterogeneity, 30% ≤ *I*^2^ < 50% indicated moderate heterogeneity, 50% ≤ *I*^2^ < 75% indicated substantial heterogeneity, and 75% ≤ *I*^2^ indicated considerable heterogeneity. Publication bias was evaluated using a funnel plot and Begg–Kendall test. Subgroup analyses based on medication status (i.e., unmedicated, medicated) were performed for levels of glutamatergic neurometabolites. To make these subgroups, a clear cut-off year for the unmedicated period was not set. If there were four or less studies on one ROI, the ROI was not included in the analysis. We used a meta-regression in mixed-effects model to assess the relationship between moderators and the effect size of glutamatergic neurometabolites between patients and controls as a dependent variable. For the meta-regression, we used “average age among subjects”, “female ratios among subjects”, or “depression severity with 17 items Hamilton Depression Rating Scale (HAMD-17)” as independent variables, because the HAMD-17 was examined in most studies as the severity scale. A mixed-model meta-regression was performed using the Comprehensive Meta-Analysis version 3. The variables for each study included: (1) clinico-demographic characteristics of the subjects (i.e., age, sex, medication status, and symptom severity, as measured by the HAMD, Beck’s Depression Inventory (BDI), or the Montgomery–Asberg Depression Rating Scale (MADRS)); (2) MRS scan methods; (3) neurometabolite quantification methods; and (4) ROIs. Given our initial assumption that Glx levels in the mPFC would be decreased in patients with depression compared with controls, the significance level for all tests was set at a *p*-value of 0.05 (two tailed).

## Results

### Characteristics of included studies

**Fig. 1:**
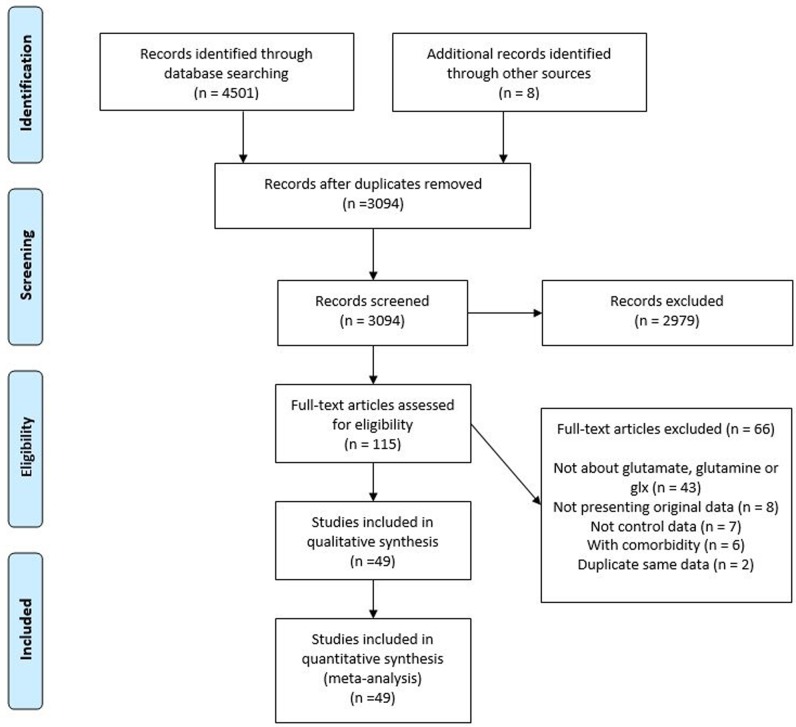
Preferred reporting items for systematic reviews and meta-analyses (PRISMA) diagram for study search

The search identified 49 studies, which included a total of 1180 patients and 1066 healthy controls. A total of 49 articles were ultimately included into our analysis (Fig. 1). Characteristics of the studies are described in Table 1. Thirty-six studies (73%) examined Glx, 27 studies (55%) examined glutamate, and 11 studies (22%) examined glutamine. Twenty-nine studies (59%) and 19 studies (39%) reported on unmedicated and medicated patients, respectively. Three studies measured glutamine levels in the DLPFC. Two studies measured Glx levels in the occipital cortex and thalamus, glutamate levels in the occipital cortex, and glutamine levels in the mTemp. One study measured glutamate levels in the thalamus and glutamine levels in the occipital cortex and thalamus. The sample sizes ranged from 9 to 63 for patients with depression and 10 to 50 for healthy controls. The average ages of each study ranged from 13.3 to 72.1 years for patients with depression and 13.6 to 72.7 years for healthy controls. Thirty-two (65%) and 12 (24%) studies were performed at a magnetic field strength of 3T and 1.5T, respectively. Magnetic resonance imaging (MRI) protocols and methodological information, including measurement technique and parameters, for each study are described in Table 1. The Newcastle-Ottawa Scale score ranged from 2 to 6 and the average was 5.1 (Supplementary Table 1), which suggests that the quality of the included studies was good on average.

**Table 1:** Characteristics of studies included for meta-analysis

Study (year)	Field strength (Tesla)	TE (ms)	TR (ms)	Acquisition sequence	CRLB threshold	Creatine scaling	Patients (*n*)	Controls (*n*)	Age	% Female	Antidepressants (yes = 1: no = 0)	Benzodiazepines (yes = 1: no = 0)	Mood stabilizers (yes = 1: no = 0)	Antipsychotics (yes = 1: no = 0)	HAMD 17	HAMD 21	HAMD 24	MADRS	BDI
Abdallah (2014) [50]	4	68	2500	J-editing	16%		23	17	43.3	72.5	0	0	0	0		30.1			
Abdallah (2015) [51]	3	68	1500				26	26	38.3	44.2	1	0	0	0	19.2				
Abdallah (2017) [18]	3	68	2500	PRESS		Cr-scaling	30	35	36.2	60.0	0	0	0	0					25.1
Auer (2000) [52]	1.5	35	2000	PRESS	20%		19	18	46.8	62.2	1	1	1	1					
Baeken (2017) [9]	3	40	2000	PRESS			18	18	46.5	66.7	0	1	0	0					
Bhagwagar (2007) [53]	3	26	3000	PRESS	20%	Cr-scaling	15	18	39.6	57.6	0	0	0	0	1.2				
Bhagwagar (2008) [12]	3	68	3000	PRESS	20%	Cr-scaling	12	11	37.6	52.2	0	0	0	0					
Binesh (2004) [54]	1.5	30	2000	L-COSY		Cr-scaling	12	16			0	0	0	0					
Block (2009) [55]	3	30,140	2000	PRESS		Cr-scaling	18	10	36.0	42.9	0	0	0	0					23.5
Brennan (2017) [56]	3	35–350	2000	JPRESS	20%	Cr-scaling	19	10	38.5	44.8	0	0	0	0	21.1			26.8	
Caetano (2005) [57]	1.5	30	6000	PRESS			14	22	13.5	36.1	1	0	0	0					
Chen (2014) [13]	1.5	30	3000	PRESS	20%		15	15	27.7	60.0	0	0	0	0	23.33				
de Diego-Adelino (2013) [58]	3	38	2000	PRESS	20%		52	16	46.1	75.0	1	1	1	1					
Gabbay (2017) [10]	3	68	1500	PRESS			44	36	16.1	57.5	0	0	0	0					24.32
Godlewska (2015) [59]	3	8500	3200	SPECIAL	20%	Cr-scaling	33	27	30.1	58.3	0	0	0	0					30.1
Godlewska (2017) [60]	7	36	3000		30%		55	50	31.3	56.0	0	0	0	0					
Grimm (2012) [61]	3	80	3000	PRESS			14	14	35.6	50.0	0				22				
Hasler (2005) [62]	3			PRESS		Cr-scaling	16	15	41.3	80.6	0								
Hasler (2007) [63]	3	68	1500	PRESS		Cr-scaling	20	20	34.4	65.0	0	0	0	0				27	
Hermens (2015) [64]	3	35		PRESS	20%	Cr-scaling	63	38	22.6	65.4	1		1	1	13.8				
Horn (2010) [65]	3	80	2000	PRESS	20%	Cr-scaling	18	22	36.4	45.0	1	0	1			17.17			
Jarnum (2011) [66]	3	30	2000	PRESS	20%		23	26	42.6	26.5	1			1	22.3				
Jayaweera (2015) [67]	3	35	2000	PRESS	20%	Cr-scaling	35	21	64.3	71.4	1	1	1	1	7.06				
Jollant (2017) [68]		8.5	3000	SPECIAL	20%	Cr-scaling	25	33	35.1	60.3	0	0	0	0			29.0		
Li (2016) [69]	3	30	1500	PRESS	20%		20	20	29.9	57.5	0	0	0	0	26.5				
Li (2014) [70]	3	80	2000	PRESS	20%	Cr-scaling	24	25	37.0	49.0	1		1			18			
McEwen (2012) [71]	3	27	3000	STEAM	20%		12	12	28.9	100.0	0	0	0	0					
Menke (2012) [72]	1.5	35	2000	PRESS	20%	Cr-scaling	61	50											
Michael (2003) [73]	1.5	20	2500	STEAM	20%		12	12	62.7	58.3	0	1	0	0				37.9	
Milne (2009) [74]	3	35	2000	PRESS	20%		28	27	37.3	54.5	1	0	0	0					19.1
Mu (2007) [75]	1.5	144	2500	PRESS		Cr-scaling	20	20	36.2	50.0	1	1	0	0			27.5		
Nery (2009) [76]	1.5	30	3000	PRESS	20%		37	40	38.4	64.9	0	0	0	0		14.8			
Pfleiderer (2003) [11]	1.5	20	2500	STEAM	20%		17	17	60.6	70.6	1	1	0	0				37.7	
Poletti (2016) [77]	3	30	2000	PRESS		Cr-scaling	19	17	39.5	69.4	1	0	0	1					
Portella (2011) [78]	3	38	2000	PRESS	30%		45	15	45.8	73.3	1	1	1	1					
Rosa (2017) [79]	3	31	1500	PRESS	20%		33	25	28.3	100.0	1				24.3				
Rosenberg (2004) [80]	1.5			PRESS			14	14	15.6	64.3	0	0	0	0					
Rosenberg (2005) [81]	1.5	30	3000	PRESS			14	14	15.6	64.3	0	0	0	0					
Sanacora (2004) [82]	2.1						33	38	38.6	52.1	0	0	0	0					
Shirayama (2017) [83]	3	30	4000	PRESS	25%		22	27	38.6	28.6	0	0	0	0	21.5				
Taylor (2009) [84]	3	26	3000	PRESS		Cr-scaling	14	16	32.2	70.0	0	0	0	0	0.3				
Taylor (2012) [85]	3	30	3000	PRESS	20%	Cr-scaling	19	27	31.8	60.9	0	0	0	0	23.2				31.5
Taylor (2017) [86]	7	10	3000	STEAM	20%		17	18	23.2	51.4	1	0	1	1				18	
Urrila (2017) [87]	3	30	2000	PRESS			9	10	16.0	0.0	0	0	0	0	11.9				15.8
Venkatraman (2009) [88]	3	30	3000	CHESS PRESS	20–30%		14	12	72.4	53.8	1							6.3	
Walter (2009) [89]	3	31–229	2500	JPRESS	20%	Cr-scaling	19	24	37.0	67.4	0	0	0	0		33.1			29.9
Yang (2016) [90]	3	30	2000	PRESS			17	11	18.5	60.7	1	1	1	1		23.94			28.65
Zhang (2016) [91]	3	69	3000	MEGA-PRESS	20%	Cr-scaling	11	11	33.9	100.0	1						22.91		
Zhao (2015) [92]	1.5	135	1700			Cr-scaling	30	30			0						23.6		

### Meta-analysis

**Fig. 2:**
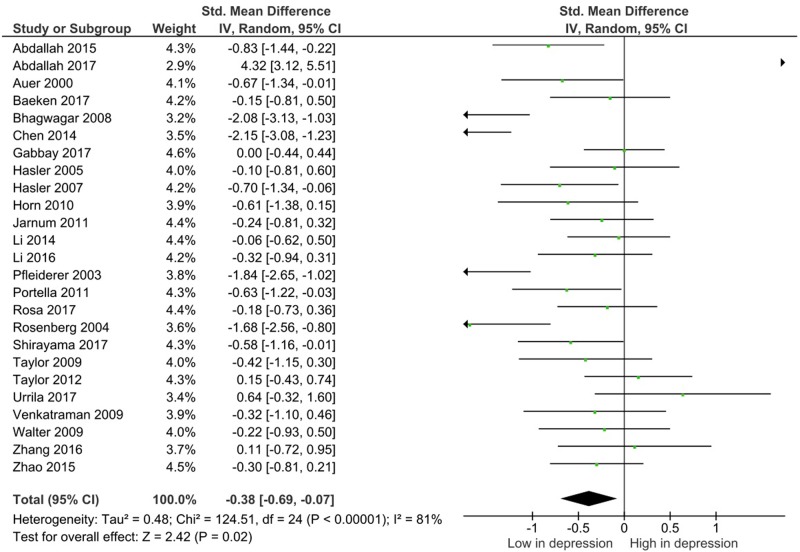
Study effect sizes of Glx differences between depression and controls in the medial prefrontal cortex. Each data marker represents a study, and the size of the data marker is proportional to the total number of individuals in that study. The summary effect size for each brain region is denoted by a diamond

**Fig. 3:**
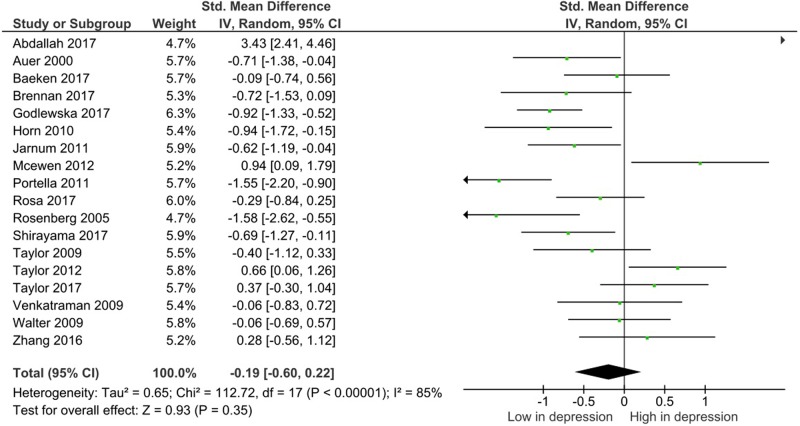
Study effect sizes of glutamate differences between depression and controls in the medial prefrontal cortex. Each data marker represents a study, and the size of the data marker is proportional to the total number of individuals in that study. The summary effect size for each brain region is denoted by a diamond

Glx levels in the mPFC were measured in 502 patients and 408 controls. There were significantly lower levels of Glx within the medial frontal cortex in patients with depression compared with controls (SMD = −0.38; 95% CI, −0.69 to −0.07; *I*^2^ = 81%; *p* = 0.2) (Table 2, Fig. 2). There were no identified differences in glutamate or glutamine levels in any regions (Table 2, Fig. 3 and Supplementary Figure 2).

**Table 2:** Meta-analysis results summary for depression and controls in all brain regions

Random effect model	Study number	Depression (*n*)	Controls (*n*)	SMD (95% CI)	*p-*Value	*I* ^*2*^
Glx						
Medial prefrontal	25	502	488	−0.38 (−0.69 to −0.07)	0.02	81%
Dorsolateral prefrontal	10	184	180	−0.36 (−0.76 to 0.04)	0.08	70%
Medial temporal	8	244	189	−0.10 (−0.48 to 0.29)	0.63	72%
Glutamate						
Medial prefrontal	18	371	359	−0.19 (−0.60 to 0.22)	0.35	85%
Dorsolateral prefrontal	7	156	161	0.13 (−0.28 to 0.55)	0.53	68%
Medial temporal	5	195	148	0.01 (−0.37 to 0.39)	0.95	64%
Glutamine						
Medial prefrontal	8	170	174	0.60 (−0.45 to 1.66)	0.26	95%

### Moderator analyses

#### Subgroup analyses and sensitivity analysis

Levels of Glx (*I*^2^ = 81%), glutamate (*I*^2^ = 85%), and glutamine (*I*^2^ = 95%) in the mPFC showed considerable heterogeneity. In addition, the leave-1-out sensitivity analysis showed that the results of Glx in the mPFC were robust. Subgroup analyses of Glx levels in the mPFC revealed that there was a significant decrease in medicated patients with depression (SMD = −0.50; 95% CI, −0.80 to −0.20; *I*^2^ = 52%; *p* = 0.001), but not in unmedicated patients (SMD = −0.27; 95% CI, −0.76 to 0.21; *I*^2^ = 87%; *p* = 0.27). The leave-1-out sensitivity analysis showed that the SMD of one study in the unmedicated depression group was high; after removing the study, there was a significant decrease in unmedicated patients with depression [18]. We also conducted subgroup analyses on mPFC Glx levels for varying reference methods. mPFC Glx levels corrected for CSF were significantly lower in patients with depression compared with controls (SMD = −0.60; 95% CI, −0.93 to −0.27; *I*^2^ = 71%; *p* < 0.001), whereas there was no significant difference in mPFC Glx levels between groups when Glx levels were referenced to creatine levels (SMD = −0.05; 95% CI, −0.63 to 0.53; I2 = 86%; *p* = 0.87).

#### Meta-regression analyses

There were no associations between subject age (coefficient: −0.0082; 95% CI: (−0.036, 0.020); *p* = 0.56), female ratio (coefficient: −0.26; 95% CI: (−2.0, 1.5); *p* = 0.77), or clinical severity of HAMD 17 (coefficient: −0.0082; 95% CI: (−0.070, 0.042); *p* = 0.62) and Glx levels in the mPFC.

### Publication bias

The Begg–Kendall test did not indicate any publication bias for mPFC Glx or mPFC glutamate, respectively (tau = −0.18, *p* = 0.21; tau = −0.19, *p* = 0.27, respectively) (Supplementary Figure 1).

## Discussion

This was the first study to compare glutamatergic neurometabolites, such as glutamate, glutamine, and Glx in broad brain regions between patients with depression and healthy controls, while also considering factors that can affect glutamatergic levels, such as medication status. We conducted a meta-analysis to compare levels of glutamatergic neurometabolites between patients with depression and controls. Our main findings are fourfold: (1) with a small effect size, Glx levels were decreased within the mPFC in patients with depression compared with controls; (2) no significant differences were found in glutamate or glutamine levels between the two groups; (3) Glx levels were lower within the mPFC in medicated patients with depression compared with controls, whereas no differences were found between unmedicated patients with depression and controls; and (4) no relationships were found between the effect sizes of mPFC Glx levels and any clinical variables in patients with depression.

### Main findings

Both animal and clinical studies have proposed that glutamatergic dysfunction is implicated in the pathophysiology of depression. Animal studies have demonstrated that stress causes depressive states that are accompanied by glutamatergic system alterations [19]. Chronic mild stress decreases the expression of NMDA receptor subunits in the frontal cortex [20, 21]. Repeated stress also decreases the expression of α-amino-3-hydroxy-5-methyl-4-isoxazolepropionic acid (AMPA) receptor subunits in the PFC [22]. Exposure to chronic stress decreases the number of mPFC neurons in rats [23, 24]. Thus, an extensive body of evidence has strongly indicated that the depressive state is related to abnormalities in the glutamatergic system.

Clinical studies have also suggested that the glutamatergic system plays an important role in the pathophysiology of depression. Some studies have reported that glutamate levels in plasma and cerebrospinal fluid (CSF) are higher in patients with depression than in controls [25–28], whereas others have indicated that there are no difference between the two groups [29, 30]. In addition, post-mortem studies have found higher glutamate levels in the mPFC of patients with depression than in that of controls [31]. However, in contrast, another study showed that there was a reduction of CSF glutamate levels in patients with treatment-resistant depression compared with controls [32]. Thus, the existing findings that have assessed glutamate levels in depression are inconsistent and seem to depend on the studies and sample source. These inconsistent findings may be due to the clinical and biological heterogeneity of depression. On the other hand, post-mortem studies have also noted glutamatergic dysfunction within the frontal cortex of patients with depression. There were observed reductions in the density of glutamatergic neurons in the orbitofrontal cortex of patients with depression [33]. In addition, reductions in the protein expression of NMDA receptor subunits (NR2A and NR2B) were observed in the PFC of patients with depression [20]. A PET study using ^11^C-ABP688 revealed lower mGluR5 availability in the PFC, cingulate cortex, insula, thalamus, and hippocampus in the depression group compared with the controls [4]. In another study, a lower mGluR5 availability with ^11^C-ABP688 was also detected in patients with depression compared with controls in many cortical areas [34]. These findings are corroborated by preclinical studies, suggesting that depressive symptoms may be associated with reductions in the mGluR5 protein [35, 36]. Collectively, these findings suggest that glutamatergic mGluR dysfunction might contribute to the pathophysiology of depression. However, the physiological implications of mGluR5 disturbances in depression still remain unclear [37–39]. Again, these observations from animal and clinical studies support the hypothesis that disruption of the glutamatergic system is associated with the pathophysiology of depression, and they further suggest that modulation of the glutamatergic system may lead to a novel therapeutic approach to depression. Our main finding of Glx reduction within the mPFC in patients with depression warrants further studies to fully elucidate the underlying mechanism of depression and the therapeutic implications of modulating the glutamatergic system.

It has been reported that a variety of depression treatments increase Glx levels in the mPFC. For example, antidepressants and electroconvulsive therapy (ECT) increase Glx levels in the mPFC in patients with depression [11, 13, 40]. In addition, several lines of investigation have shown that ketamine, an NMDA receptor antagonist, has antidepressant effects and increases glutamate levels in the PFC in patients with depression through NMDAreceptor inhibition and subsequent AMPAR activation [41, 42]. The NMDAreceptor inhibition and subsequent AMPAreceptor stimulation leads to inhibition of eukaryotic elongation factor 2 kinase, as well as activation of brain-derived neurotrophic factor, tropomyosin-related kinase B, and mammalian target of rapamycin signaling, thereby increasing levels of synaptic proteins in the PFC [43–45]. Overall, these results support hypo-glutamatergic function in depression and are in line with our finding of decreased levels of Glx within the mPFC in patients with depression.

### Findings of moderator analyses

The main finding should be confirmed in further studies because the heterogeneity of the included studies was high. Our subanalyses found that Glx levels were lower in the mPFC of medicated patients with depression than those of controls, whereas there were no differences between unmedicated patients with depression and controls. However, one study reported high Glx levels in unmedicated patients compared with controls [18]. After removing this study, we found that there was also a significant decrease of Glx levels in unmedicated patients with depression in comparison with controls. In addition, effects sizes were similar between unmedicated and medicated patients compared with controls. However, as described above, it has previously been reported that treatment of depression is associated with Glx elevations within the mPFC in patients with depression; this has been shown with antidepressant treatment, ECT, and ketamine administration [11, 13, 40, 42]. It is difficult to accurately assess the difference between the treatment group and the untreated group due to the high heterogeneity of the included studies in both groups. Furthermore, medication information other than antidepressants, such as benzodiazepines, mood stabilizers, and antipsychotics, was not sufficient to perform the meta-regression analyses. Thus, further research is clearly needed to elucidate the effects of various depression treatments on glutamatergic neurometabolites.

Notably, the present meta-regression did not find that glutamatergic neurometabolite concentrations in patients vary in association with symptom severity. Thus, the group difference in mPFC Glx levels between patients with depression and controls could not be explained by symptom severity. Of note, the symptomatology of depression, including psychotic symptoms or melancholic types, represents a clinically important factor that might also contribute to the heterogeneity. However, information concerning symptoms was insufficient in the included studies for further analyses. Thus, further studies will be required to investigate specific subtypes of depression.

Our subgroup analyses found that mPFC Glx levels corrected for CSF were lower in patients with depression compared with controls, whereas there were no group differences in mPFC Glx levels referenced to creatine levels. Given that creatine levels are lower in patients with depression compared with controls (Supplementary Figure 3), Glx levels referenced to creatine could be overestimated in patients with depression. Taken together, these findings again suggested that mPFC Glx levels may be decreased in patients with depression in comparison with controls.

### Limitations

This meta-analysis has some limitations. First, although the meta-analysis analyzed data region by region, both voxel sizes and ROIs were different among the included studies. The quality control procedure also varied among studies. These differences might skew the results of ROI analyses. Second, the field strength of most studies was 3T or lower, which precluded the accurate division of glutamate and glutamine. We could not specify which glutamatergic metabolites in which cell types are affected in depression. Glx is made up of both glutamate and glutamine but glutamate accounts for about 80% of Glx levels at 1.5T or 3T [46]. ^1^H MRS also does not provide information regarding cell types. Future studies should include studies with increased field strength to improve separation of individual signal spectra. Third, the present study did not consider confounders such as food or smoking status. The majority of studies included in the meta-analysis did not include meal or smoking information. Food intake can influence Glx levels in the brain, with Glx decreasing by as much as 17% in the PFC after fasting [47]. Smoking also interferes with the glutamate levels in the mPFC [48]. Thus, studies that report food or smoking condition should be included in future meta-analyses. Fourth, the number of included subjects in studies was relatively small. The sample sizes ranged from 9 to 63 for depression and 10–50 for healthy controls. Further studies with larger sample sizes should be conducted. Fourth, we could not analyze specific age groups even though we included studies with no exclusion in age. Indeed, a few studies investigated pediatric subjects. Thus, future studies are warranted to investigate a wide range of age groups in an effort to confirm whether the results would be consistent in both pediatric and adult patients with depression. Finally, statistical heterogeneity is high in terms of Glx in the mPFC. In the past meta-analysis, mPFC Glx levels in patients with depression were associated with symptom severity in meta-regression analyses, although such a relationship was not identified in our analysis [15]. This is likely attributable to the clinical heterogeneity caused by differences in patient characteristics [49] or the influence of other methodological factors such as neurometabolite reference method (i.e., Cr versus water).

## Conclusion

The results of this meta-analysis suggest that depression is associated with decreased levels of Glx in the mPFC. This further substantiates the development of novel treatment interventions that seek to modulate the glutamatergic system in patients with depression.

## Electronic supplementary material

supplementary tables and supplementary figure legends

Supplementary Figure 1A

Supplementary Figure 1B

Supplementary Figure 2A

Supplementary Figure 2B

Supplementary Figure 2C

Supplementary Figure 2D

Supplementary Figure 2E

Supplementary Figure 3
