# Supplementary material for: Glutamatergic neurometabolite levels in major depressive disorder: a systematic review and meta-analysis of proton magnetic resonance spectroscopy studies
Source: Mol Psychiatry. 2018 Oct 12;24(7):952–64. doi: 10.1038/s41380-018-0252-9 (PMC6755980; doi:10.1038/s41380-018-0252-9)
Supplement: Supplementary file 1 — supplementary tables and supplementary figure legends [file 41380_2018_252_MOESM1_ESM.docx]

Supplementary Figure 1. Funnel plot of Glx differences in the medial prefrontal cortex (A) and glutamate differences in the medial prefrontal cortex (B)

Supplementary Figure 2. Study effect sizes of neurometabolites differences between depression and controls

Each data marker represents a study, and the size of the data marker is proportional to the total number of individuals in that study. The summary effect size for each brain region is denoted by a diamond. (A) Glx for the dorsolateral prefrontal cortex; (B) Glx for the medial temporal cortex; (C) Glutamate for the dorsolateral prefrontal cortex; (D) Glutamate for the medial temporal cortex; (E) Glutamine for the medial prefrontal cortex

Supplementary Figure 3. Study effect sizes of creatine level differences between patients and controls

Effect sizes of studies that measured creatine level differences between patients with depression and controls in all brain regions. Each data marker represents a study, and the size of the data marker is proportional to the total number of individuals for each study. The summary of effect size for each brain region is denoted by a diamond.

Supplementary Table 1 The Newcastle-Ottawa Scale

|  | Selection |  |  |  | Comparability |  |  |
| --- | --- | --- | --- | --- | --- | --- | --- |
| Study and Year | Case difinition | Representativeness | Selection of Control | Definition of Control | Age | Sex | Total |
| Abdallah CG (2014) | 1 | 1 | 1 | 1 | 1 | 1 | 6 |
| Abdallah CG (2015) | 1 | 1 | 1 | 1 | 1 | 1 | 6 |
| Abdallah CG (2017) | 1 | 0 | 1 | 1 | 1 | 1 | 5 |
| Auer DP (2000) | 1 | 0 | 1 | 1 | 0 | 1 | 4 |
| Baeken C (2017) | 1 | 0 | 1 | 1 | 1 | 1 | 5 |
| Bhagwagar Z (2007) | 1 | 1 | 1 | 1 | 1 | 1 | 6 |
| Bhagwagar Z (2008) | 1 | 1 | 1 | 1 | 0 | 0 | 4 |
| Binesh N (2004) | 1 | 1 | 0 | 0 | 1 | 0 | 3 |
| Block W (2009) | 1 | 1 | 1 | 1 | 1 | 1 | 6 |
| Brennan B (2017) | 1 | 1 | 1 | 1 | 1 | 1 | 6 |
| Caetano S (2005) | 1 | 0 | 1 | 1 | 1 | 1 | 5 |
| Chen LP (2014) | 1 | 1 | 1 | 1 | 1 | 1 | 6 |
| De Diego-Adeliño J (2013) | 1 | 1 | 1 | 1 | 0 | 0 | 4 |
| Gabbay V (2017) | 1 | 0 | 1 | 1 | 1 | 1 | 5 |
| Godlewska BR (2015) | 1 | 1 | 1 | 1 | 1 | 1 | 6 |
| Grimm S (2012) | 1 | 1 | 1 | 1 | 1 | 1 | 6 |
| Hasler G (2005) | 1 | 1 | 1 | 1 | 1 | 1 | 6 |
| Hasler G (2007) | 1 | 1 | 1 | 1 | 1 | 1 | 6 |
| Hermens DF (2015) | 1 | 1 | 1 | 1 | 1 | 1 | 6 |
| Horn DI (2010) | 1 | 1 | 1 | 1 | 1 | 1 | 6 |
| Järnum H (2011) | 1 | 1 | 1 | 1 | 1 | 1 | 6 |
| Jayaweera HK (2015) | 1 | 1 | 0 | 0 | 1 | 1 | 4 |
| Jollant F (2017) | 1 | 1 | 1 | 1 | 0 | 1 | 5 |
| Li H (2016) | 1 | 1 | 1 | 1 | 1 | 1 | 6 |
| Li M (2014) | 1 | 1 | 1 | 1 | 1 | 1 | 6 |
| McEwen AM (2012) | 1 | 0 | 1 | 1 | 1 | 1 | 5 |
| Menke A (2012) | 1 | 1 | 1 | 1 | 1 | 1 | 6 |
| Michael N (2003) | 1 | 1 | 0 | 0 | 1 | 1 | 4 |
| Milne A (2009) | 1 | 1 | 1 | 1 | 1 | 1 | 6 |
| Mu J (2007) | 1 | 1 | 1 | 0 | 1 | 1 | 5 |
| Nery FG (2009) | 1 | 1 | 1 | 1 | 1 | 1 | 6 |
| Pfleiderer B (2003) | 1 | 1 | 1 | 1 | 1 | 1 | 6 |
| Poletti S (2016) | 1 | 1 | 0 | 0 | 0 | 0 | 2 |
| Portella MJ (2011) | 1 | 1 | 1 | 1 | 1 | 0 | 5 |
| Rosa C (2017) | 1 | 0 | 0 | 1 | 1 | 1 | 4 |
| Rosenberg D (2004) | 1 | 1 | 1 | 1 | 1 | 1 | 6 |
| Rosenberg D (2005) | 1 | 1 | 1 | 1 | 1 | 1 | 6 |
| Sanacora G (2004) | 1 | 1 | 1 | 1 | 0 | 0 | 4 |
| Shirayama Y(2017) | 1 | 1 | 0 | 0 | 1 | 1 | 4 |
| Taylor MJ (2009) | 1 | 0 | 0 | 0 | 1 | 1 | 3 |
| Taylor MJ (2012) | 1 | 1 | 1 | 0 | 1 | 1 | 5 |
| Taylor R (2017) | 1 | 1 | 1 | 1 | 1 | 0 | 5 |
| Urrila A (2017) | 1 | 1 | 1 | 1 | 1 | 1 | 6 |
| Venkatraman TN (2009) | 1 | 1 | 1 | 1 | 1 | 1 | 6 |
| Walter M (2009) | 0 | 1 | 1 | 1 | 1 | 1 | 5 |
| Yang XR (2016) | 1 | 1 | 1 | 0 | 1 | 0 | 4 |
| Zhang X (2016) | 1 | 1 | 1 | 0 | 1 | 1 | 5 |
| Zhao L (2015) | 1 | 1 | 1 | 1 | 1 | 1 | 6 |
