# Supplementary figures and images for: Glutamatergic neurometabolite levels in major depressive disorder: a systematic review and meta-analysis of proton magnetic resonance spectroscopy studies
Source: Mol Psychiatry. 2018 Oct 12;24(7):952–64. doi: 10.1038/s41380-018-0252-9 (PMC6755980; doi:10.1038/s41380-018-0252-9)

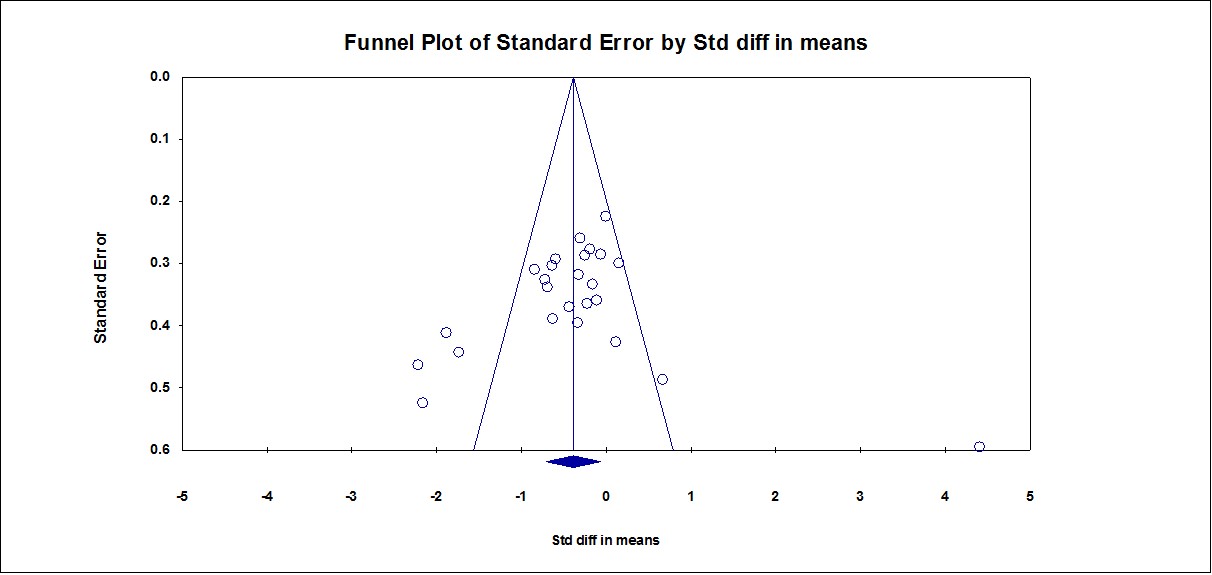

Supplement: Supplementary file 2 — Supplementary Figure 1A [file 41380_2018_252_MOESM2_ESM.jpg]

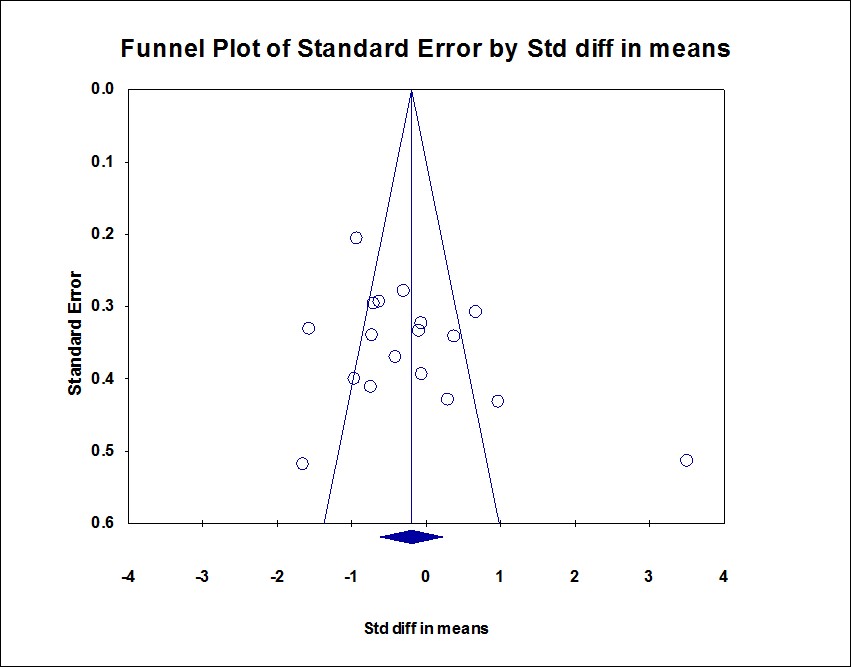

Supplement: Supplementary file 3 — Supplementary Figure 1B [file 41380_2018_252_MOESM3_ESM.jpg]

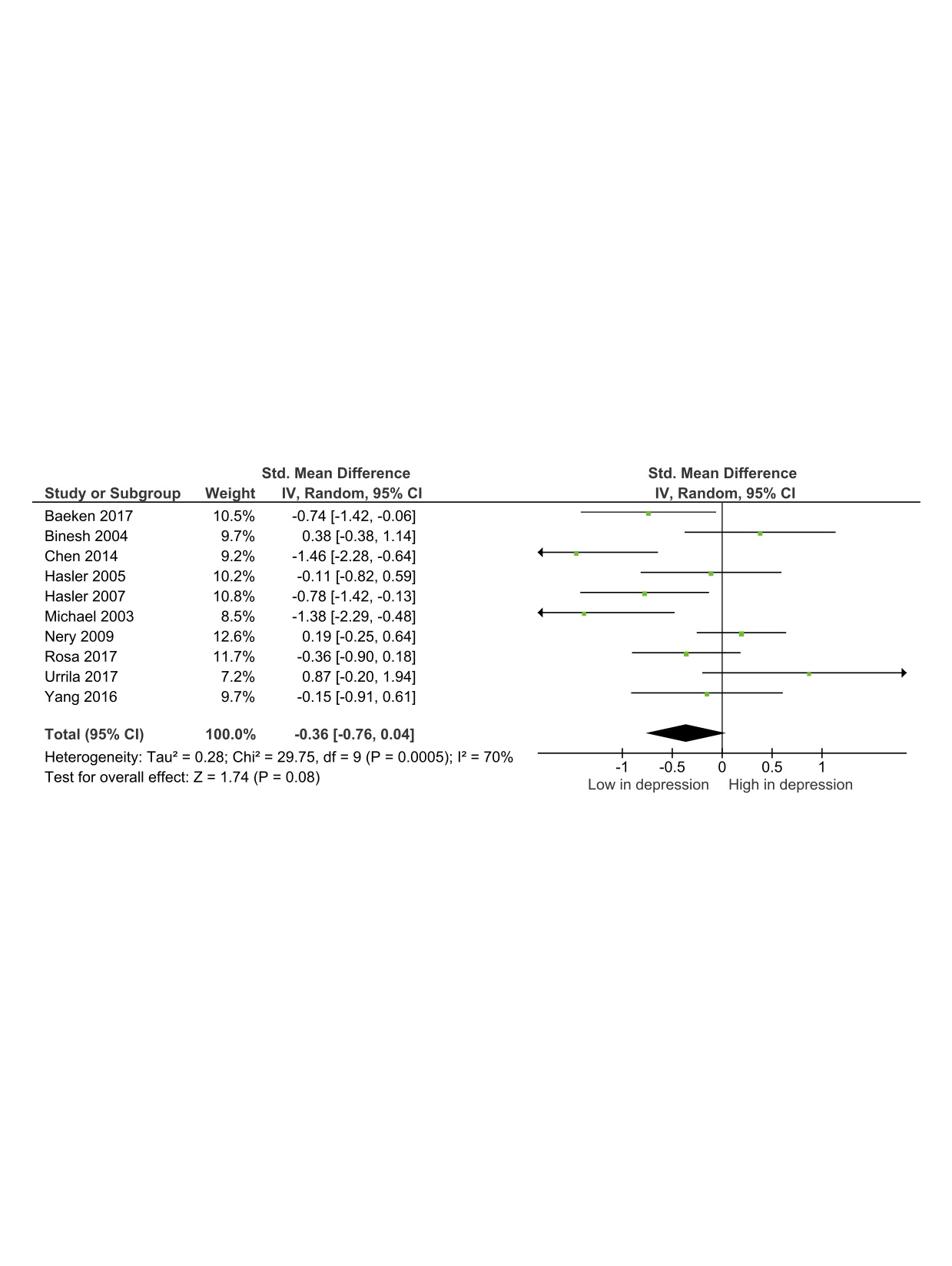

Supplement: Supplementary file 4 — Supplementary Figure 2A [file 41380_2018_252_MOESM4_ESM.jpg]

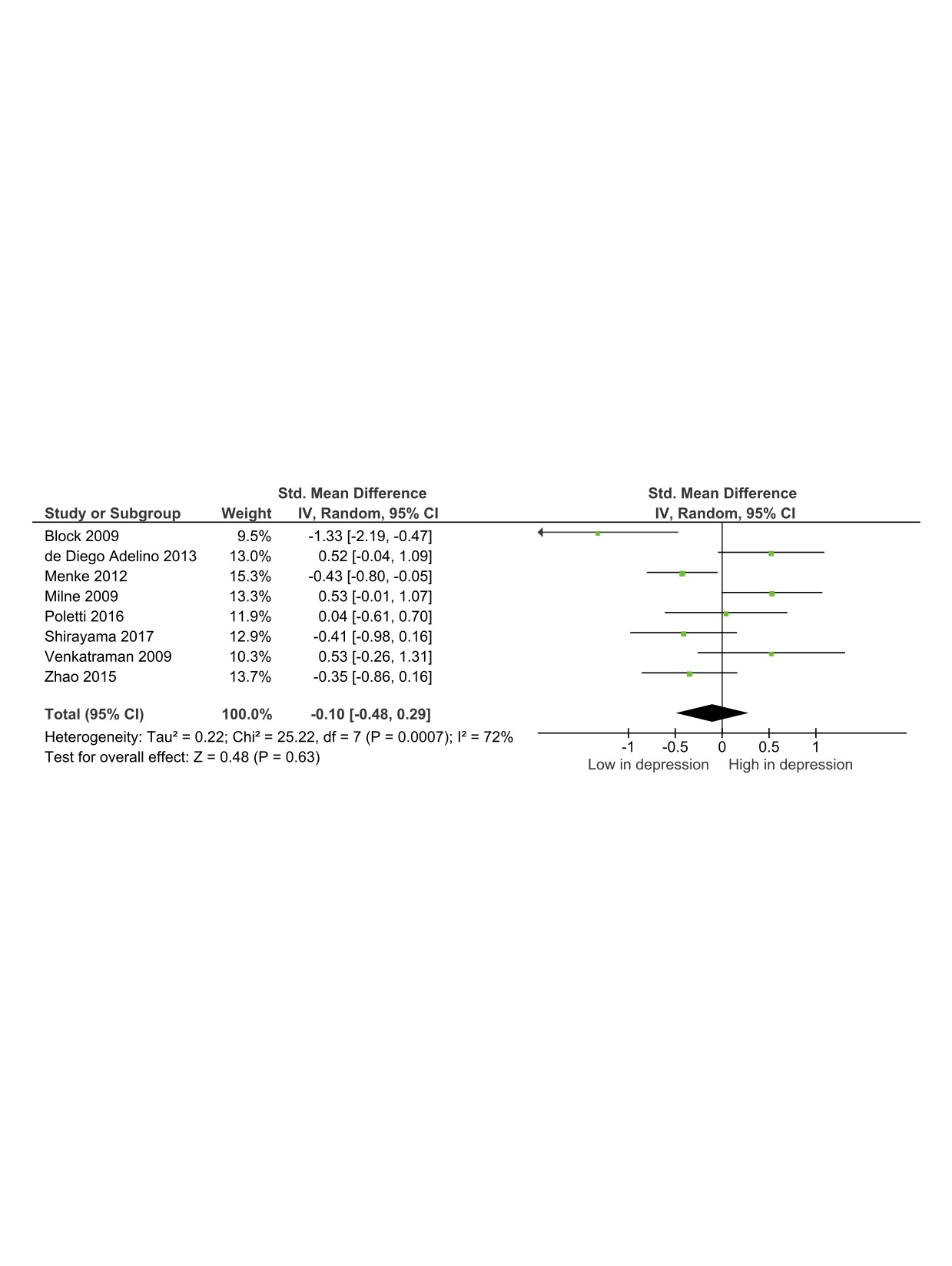

Supplement: Supplementary file 5 — Supplementary Figure 2B [file 41380_2018_252_MOESM5_ESM.jpg]

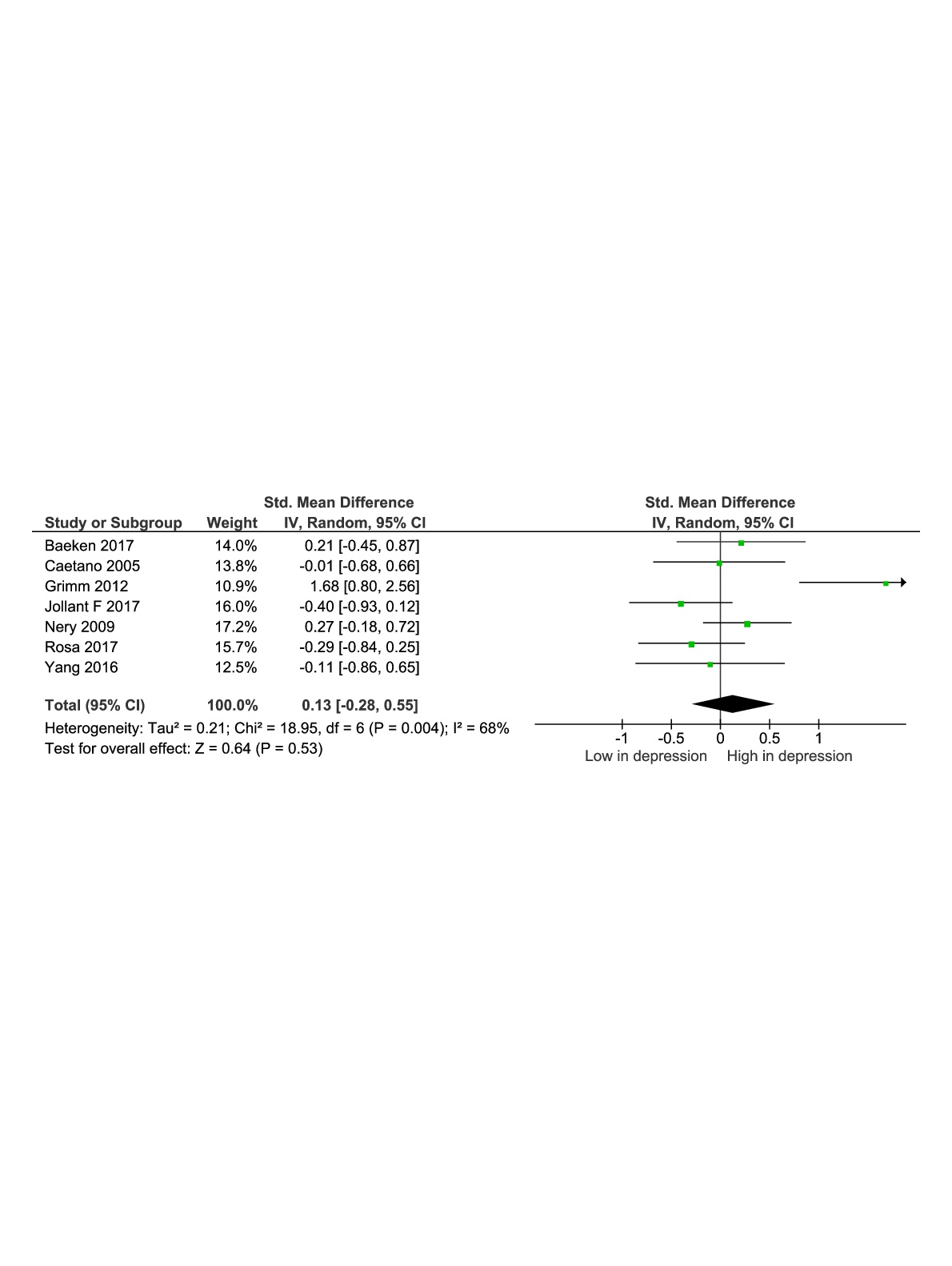

Supplement: Supplementary file 6 — Supplementary Figure 2C [file 41380_2018_252_MOESM6_ESM.jpg]

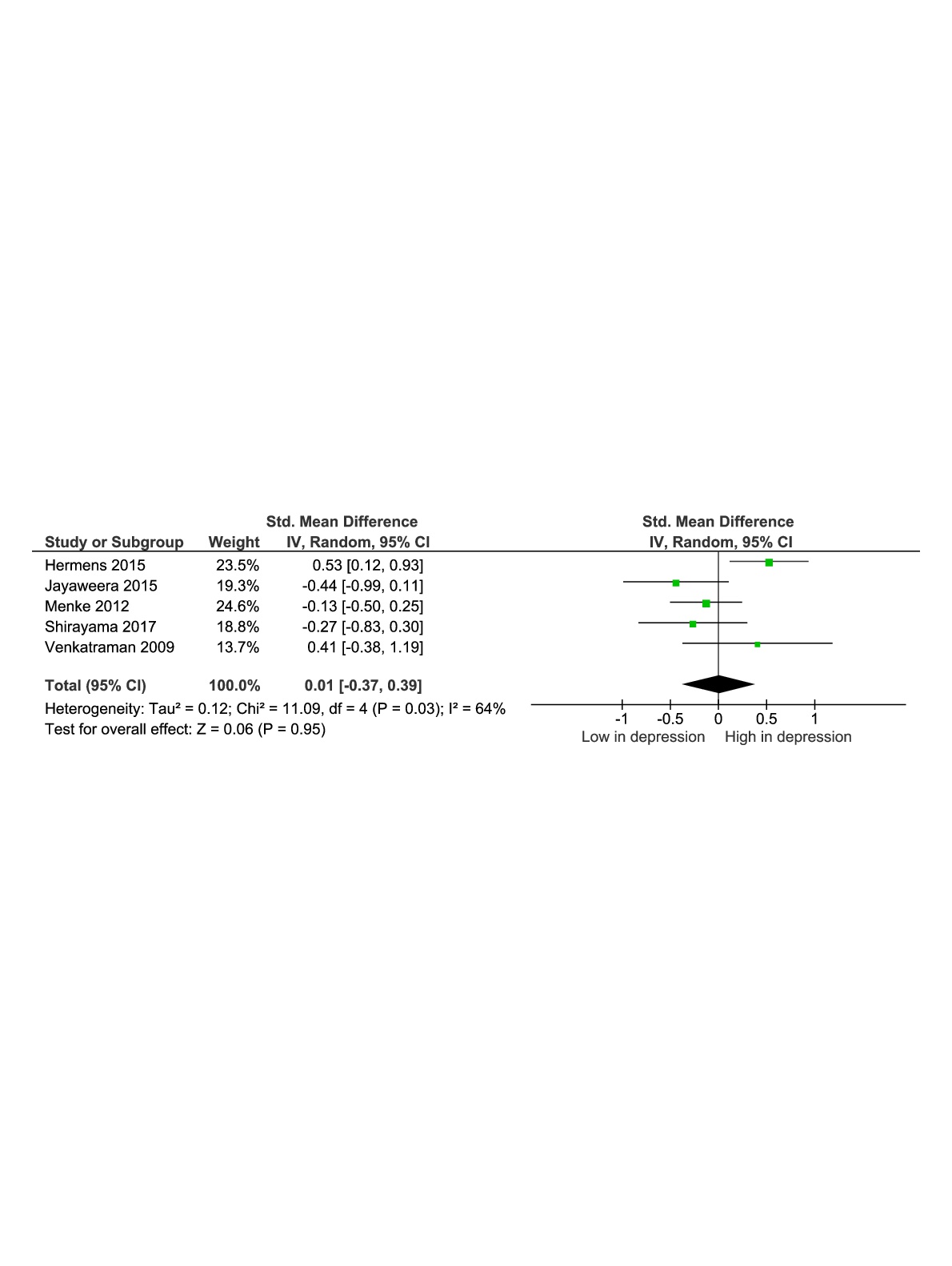

Supplement: Supplementary file 7 — Supplementary Figure 2D [file 41380_2018_252_MOESM7_ESM.jpg]

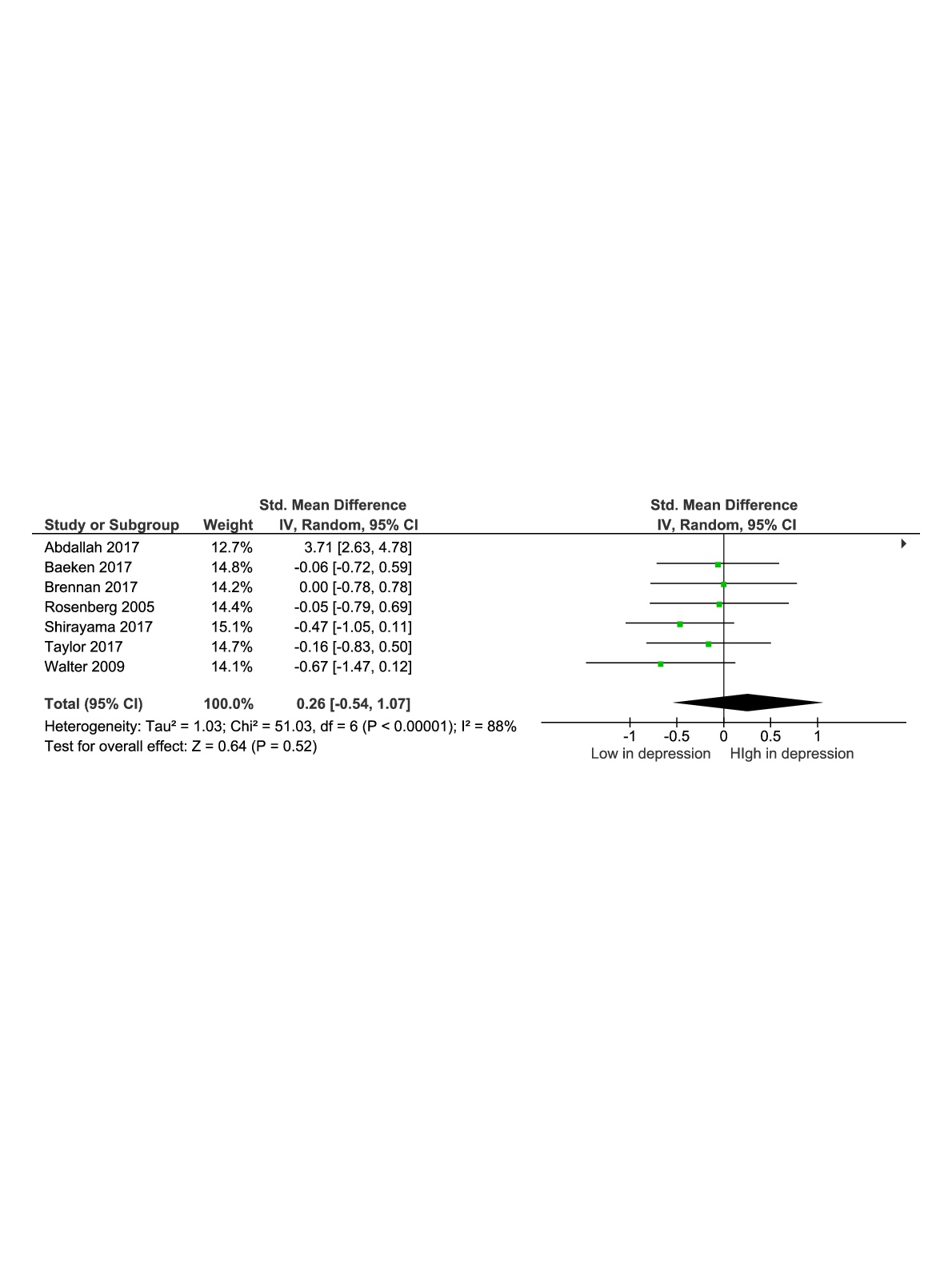

Supplement: Supplementary file 8 — Supplementary Figure 2E [file 41380_2018_252_MOESM8_ESM.jpg]

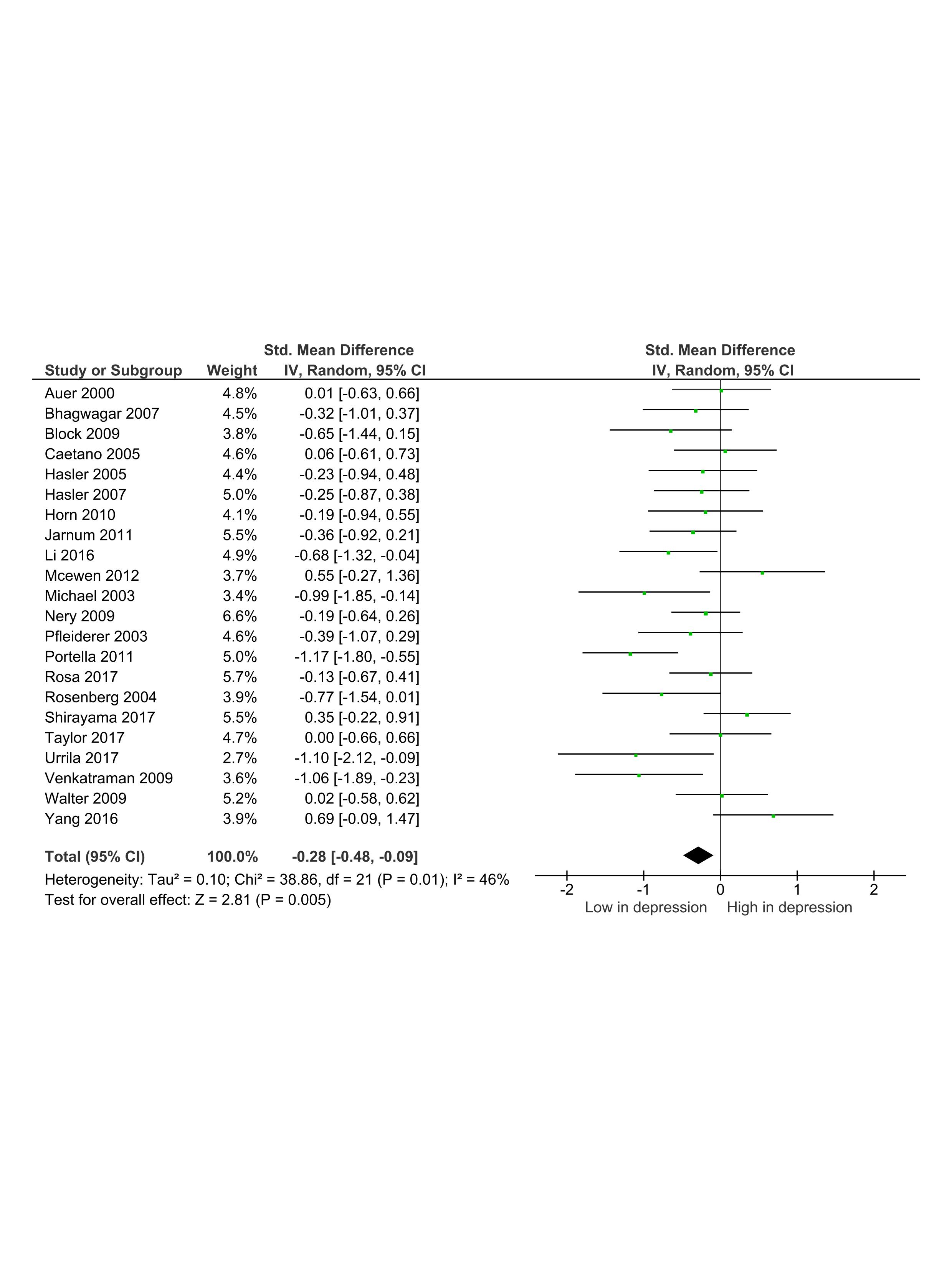

Supplement: Supplementary file 9 — Supplementary Figure 3 [file 41380_2018_252_MOESM9_ESM.jpg]
